# Glucose alarms approach with flash glucose monitoring system: a narrative review of clinical benefits

**DOI:** 10.1186/s13098-025-01797-3

**Published:** 2025-07-16

**Authors:** Marcio Krakauer, Solange Travassos, Melanie Rodacki, Monica A. L. Gabbay, André G. D. Vianna, Mauro Scharf, Rodrigo N. Lamounier, Denise Reis Franco, Levimar Rocha Araújo, Luis Eduardo Calliari

**Affiliations:** 1 Sociedade Brasileira de Diabetes, São Paulo, Brazil; 2 Internal Medicine Department, Universidade Federal do Rio de Janeiro, Rio de Janeiro, Brazil; 3 Diabetes Center, São Paulo Federal University, São Paulo, Brazil; 4 Department of Endocrine Diseases, Hospital Nossa Senhora das Graças, Curitiba Diabetes Centre, Curitiba, Brazil; 5 School of Medicine, Internal Medicine Department, Federal University of Minas Gerais (UFMG) Brazil, Belo Horizonte, Brazil; 6 CPCLIN-Centro de Pesquisas Clínicas–DASA, São Paulo, Brazil; 7 Minas Gerais School of Medical Sciences, Belo Horizonte, Minas Gerais Brazil; 8 Department of Pediatrics, Santa Casa School of Medical Sciences, Sao Paulo, Brazil

**Keywords:** Glucose alarm, Flash continuous glucose monitoring, Diabetes, Hyperglycemia, Hypoglycemia

## Abstract

Attaining an adequate glycemic control has been associated with a better prognosis and with a reduction in the risk of developing long-term microvascular and macrovascular diabetic complications. Continuous glucose monitoring (CGM) has been shown to improve glycemic control and reduce blood glucose variability. Furthermore, CGM is associated with greater treatment adherence and higher satisfaction. Hypoglycemia is the most frequent acute complication in individuals with insulin treated diabetes and may limit the achievement of glycemic control. Furthermore, repeated episodes of hypoglycemia, particularly when a severe hypoglycemia event occurs are associated with adverse outcomes. The introduction of glucose alarms improves not only safety of subjects, but also contributes to improve glycemic control. However, depending on the glycemic thresholds, the frequency of alarms could be perceived as excessive, leading to a state of ‘alarm fatigue’, limiting the effective response to the alarms by the individual. The optimization of alarm thresholds tailored to individual needs and preferences can enhance the clinical utility of CGM while minimizing alarm fatigue. When alarms occur, their underlying causes should be investigated to enable appropriate corrections and adjustments. CGM systems equipped with alarms, such as FreeStyle Libre 2, have demonstrated efficacy in reducing hyperglycemia and severe hypoglycemic events, leading to improvements in time in range and quality of life of people with diabetes.

## Background

Attaining an adequate glycemic control in people with diabetes has been associated with a better prognosis and with a reduction in the risk of developing long-term microvascular and macrovascular diabetic complications [1, 2]. Classically the determination of hemoglobin A1c (HbA1c) levels has been used for assessing glycemic control, but HbA1c cannot detect the short-term variations of glucose levels, the time the subject is in hypoglycemia or hyperglycemia, including asymptomatic or severe hypoglycemic events, or glycemic variability [3]. Self-monitoring of blood glucose provides an accurate measure of capillary glucose concentrations, and it has been shown that the higher the frequency of self-monitoring of blood glucose (SMBG) measurements, the better the HbA1c [4]. However, self-monitoring of blood glucose does not sufficiently detect glycemic variability and trends, especially during the night or in case of asymptomatic hypoglycemia and it can be inconvenient, painful and difficult to maintain long-term treatment [5–7].

Continuous Glucose Monitoring (CGM) measures real-time interstitial glucose levels, enabling early detection of hypo- and hyperglycemia and improves glycemic control [3, 8]. CGM provides information about metrics that are relevant to glucose control [8, 9], such as time in range (TIR—time spend with glucose values between 70 and 180 mg/dL) [10], time above range (TAR—time spend with glucose values higher than 180 mg/dL) and time below range (TBR—time spend with glucose values lower than 70 mg/dL), coefficient of variation [11], and glucose monitoring index (GMI) [12]. Briefly, TIR refers to the amount of time you spend in the target blood glucose range and is marker for risk of chronic complications; coefficient of variation is calculated as the standard deviation of sensor glucose values during the observation period divided by the mean of sensor glucose values in the same observation period and reflects glucose variability. GMI is an estimate of the laboratory HbA1c level based on the average glucose measured via CGM [10–12]. Lastly, time in tight range (TITR) is defined as the percentage of time spent in the target glucose range of 70–140 mg/dL, and represents a promising novel CGM glycemic metric [13, 14].

Compared to SMBG, use of CGM has shown to improve glycemic control (HbA1c and TIR) and decrease glucose variability. Moreover, CGM is also associated with higher treatment adherence and satisfaction of individuals [15–19] and reduce of hospitalization [18, 20–24]. According to all this information, CGM devices are considered the standard of care for most subjects with type 1 diabetes (T1D) [8, 25], but also for those individuals with type 2 diabetes that receive intensive treatment with insulin, and even for those with type 2 diabetes that do not achieve the recommended targets despite basal insulin-only regimens or other antidiabetic drugs, or when minimizing hypoglycemic risk is warranted [26–28].

In general, the term “CGM systems” refers to both intermittently scanned continuous glucose monitoring (isCGM) and real-time CGM (rtCGM) [8]. The isCGM sensors measure and store glucose levels continuously but require scanning for visualization of glucose values. Therefore, the user needs an active attitude to visualize glucose levels, and, as expected, the higher frequency of daily scans is related to better glycemic control [29, 30]. The rtCGM measure and display glucose levels continuously to the users’ smartphone or reader. Both systems detect hypoglycemia or hyperglycemia, have frequent measurements and trend arrows, but the alarm is only present in the rtCGM or in the isCGM with optional alarms feature, allowing the individuals to respond faster to prevent or treat these events (e.g., eating a meal, modifying insulin dose, etc.) [8, 31]. The term iCGM refers to integrated CGM (iCGM), which are systems that meet specific and rigorous requirements of the FDA with published performance data verifying accuracy that are used to directly inform decision-making in the treatment of diabetes (i.e., insulin dosing) [32].

CGM and SMBG both provide a single “point-in-time” glucose measurement [33]. Beyond that, CGM sensors transmit additional information to the user, including a trend arrow indicating how glucose levels are changing, as well as a trend graph visually displaying the person’s glucose concentrations [33].

Hypoglycemia is the most frequent acute complication in people with type 1 diabetes and may limit the attainment of a good glycemic control. Furthermore, repeated episodes of hypoglycemia, particularly when a severe hypoglycemia event occurs are associated with adverse outcomes [34, 35]. Although CGM has been shown to reduce time spent in hypoglycemia, early detection remains essential for timely interventions and the prevention of further complications [15, 36, 37].

In this context, isCGM and rtCGM devices with optional glucose alarms are becoming available worldwide. Setting up glucose threshold alerts may warn about potentially unwanted glycemic conditions (i.e., hypoglycemic and hyperglycemic events). These alerts are beneficial, since they are important for attaining glycemic control, reducing the risk of hypoglycemia, as subjects can react sooner than without alerts [38–40]. Despite the fact that the use of alarms in CGM systems provide relevant information for improving glycemic control, and reducing the risk of potential complications, optimal settings of alarms in CGM are not clearly established and should be individualized according to the clinical characteristics of the individuals [41–44].

## Glucose alarms

The introduction of glucose alarms not only improves the safety of people with diabetes (i.e., high alarms to reduce the risk of dangerously high glucose; low alarms to reduce the risk of severe hypoglycemia), but alarms also can be used to increase TIR (i.e., high alarms to reduce time above range; low alarms to reduce time below range) [39, 45]. However, depending on the glycemic thresholds, the frequency of alarms could be perceived as excessive, leading to a state of ‘alarm fatigue’, limiting the effective response to the alarms by the individuals [46, 47]. Importantly, CGM-specific education is crucial to enhance the regular use of glycemic alarms [44, 48]. Therefore, it is relevant to define both, the hypo- and hyperglycemia thresholds (i.e., elevated low alarm may be appropriate when the primary goal is to reduce the risk of hypoglycemia and glucose variability, but accepting certain degree of mild hyperglycemia and more alarms will occur when glucose levels are safe and stable; by contrast, a low hyperglycemia alarm threshold provides a tighter glycemic control, but with higher frequency of hypoglycemic events [39]. The quantity of alarms may depend on a number of factors, including how often glucose is desirable to be checked, the glucose variability, the awareness of hypoglycemia, or the level of low glucose alarm is set, considering that the higher the alarm is set, the more alarms occur [45–47, 49, 50].

There are specific guidelines regarding CGM glucose alarms, such as the Brazilian Diabetes Society [51] and Diabetes Technology Network (DTN-UK) [50]. They both recommended that individuals who are new to CGM typically do not activate the high glucose alarm during the initial days and instead focus on familiarizing themselves with the system, as well as glucose levels improve, the glucose alarms could be gradually changed [52].

### Low glucose alarm

Hypoglycemia is one of the most relevant limiting factors for the management of diabetes. Severe hypoglycemia, and nocturnal hypoglycemia may be particularly dangerous for people with diabetes. In fact, severe hypoglycemia, which occurs at nighttime in more than half of individuals, can increase the risk of death and microvascular complications [53, 54]. In this context, effective low glucose alarm may be essential for the early detection and prevention of severe hypoglycemia [39, 55]. This would not only improve glycemic parameters (mean, variability, TIR, time below range), but more importantly, the long-term consequences of diabetes, and the quality of life of people with diabetes, including the fear of hypoglycemia [56].

The individuals that may benefit more from low glucose alarms include: pregnant woman with diabetes, which has a higher risk of hypoglycemia since tighter glycemic control is desired, and consequently more intensive treatment is required (see below in the section pregnancy);; children, since hypoglycemia may not be clearly recognized; older person classified as complex/intermediate health or very complex/poor health; people with asymptomatic, oligosymptomatic and/or nocturnal hypoglycemia; and subjects with a previous episode of severe hypoglycemia [56].

When setting the low glucose alert threshold, it is important to determine whether the alert should serve as a 'safety net' to detect missed events or provide warnings for all low glucose episodes to minimize the risk of potential events. On the other hand, age is an important aspect do be considered to set the low glucose threshold [51]. Thus, in younger (< 6 years) and older frail subjects, a higher threshold between 70 and 90 mg/dL may be beneficial, whereas in the others, a lower threshold between 70 and 80 mg/dL could be considered [51]. Those with frequent nocturnal hypoglycemia may benefit from setting higher low glucose alarm limits at night. In general, most individuals may have 1–2 events < 70 mg/dL per day, that implies 1–2 low alarms per day. Modifying this threshold implies a change in the number of daily alerts [51].

**Fig. 1:**
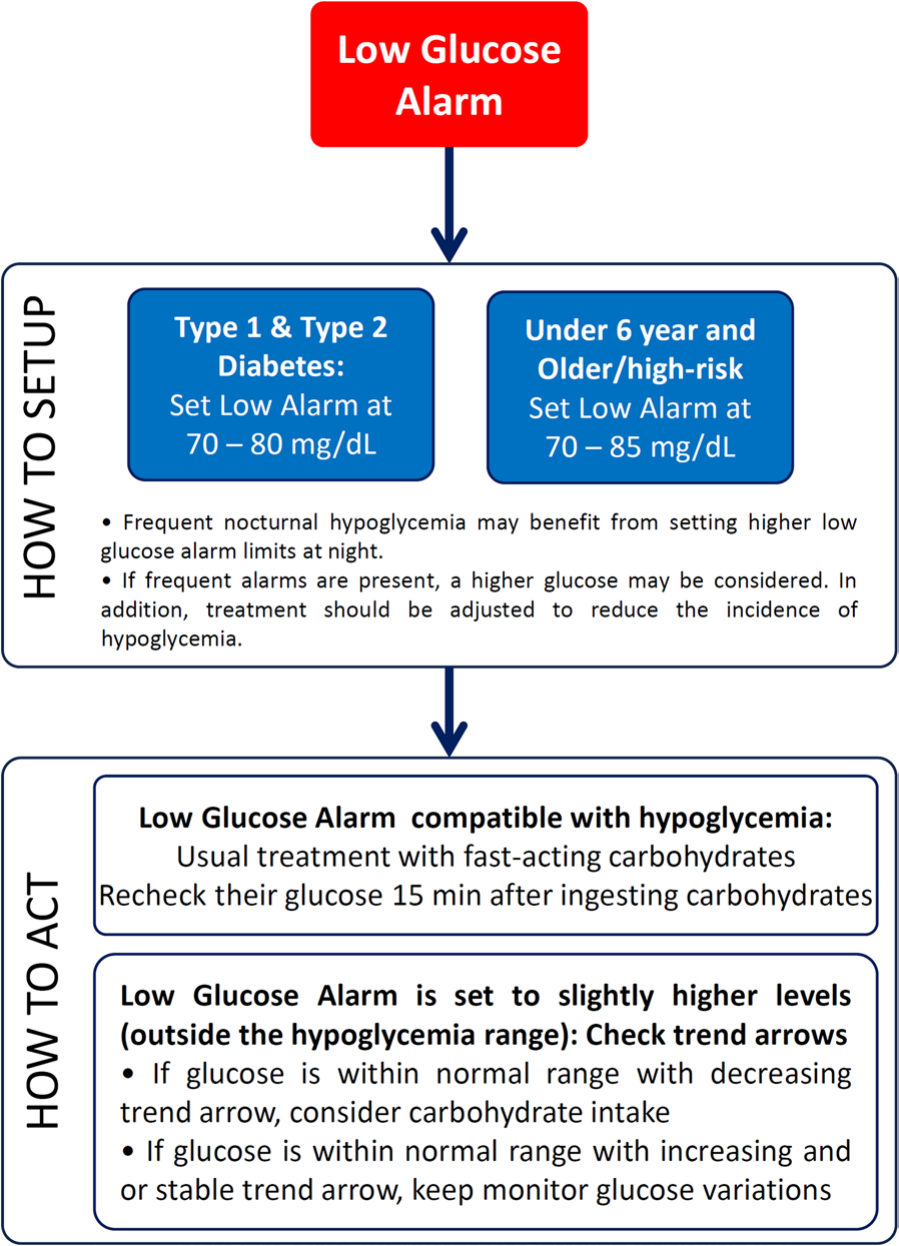
How to set up Low glucose alert threshold. Based on reference [51]

Although lowering the low glucose alert level could reduce the number of alarms, before doing this, it should be ascertained why the subject is receiving these alarms. If frequent alarms are present, a higher glucose may be considered. In addition, treatment should be adjusted to reduce the incidence of hypoglycemia. In this context, before considering changing low glucose thresholds, it should be analyzed whether there is a pattern that can predict and prevent alerts by adjusting the insulin/carbohydrate ratio, or if it is possible to predict and prevent the event by acting earlier [48, 54]. A proposal of algorithm about how to set the low glucose alarms in FreeStyle Libre 2 system, according to the clinical characteristics of subjects is shown in Fig. 1 [51].

### High glucose alarm

The use of high glucose alerts is associated with better glycemic control [57]. Individuals that may benefit more from high glucose alarms include pregnant woman with diabetes that require a tight glycemic control, children at risk for diabetic ketoacidosis, elderly with comorbidities, users of continuous insulin infusion systems and among subjects that require improving TIR [56].

**Fig. 2:**
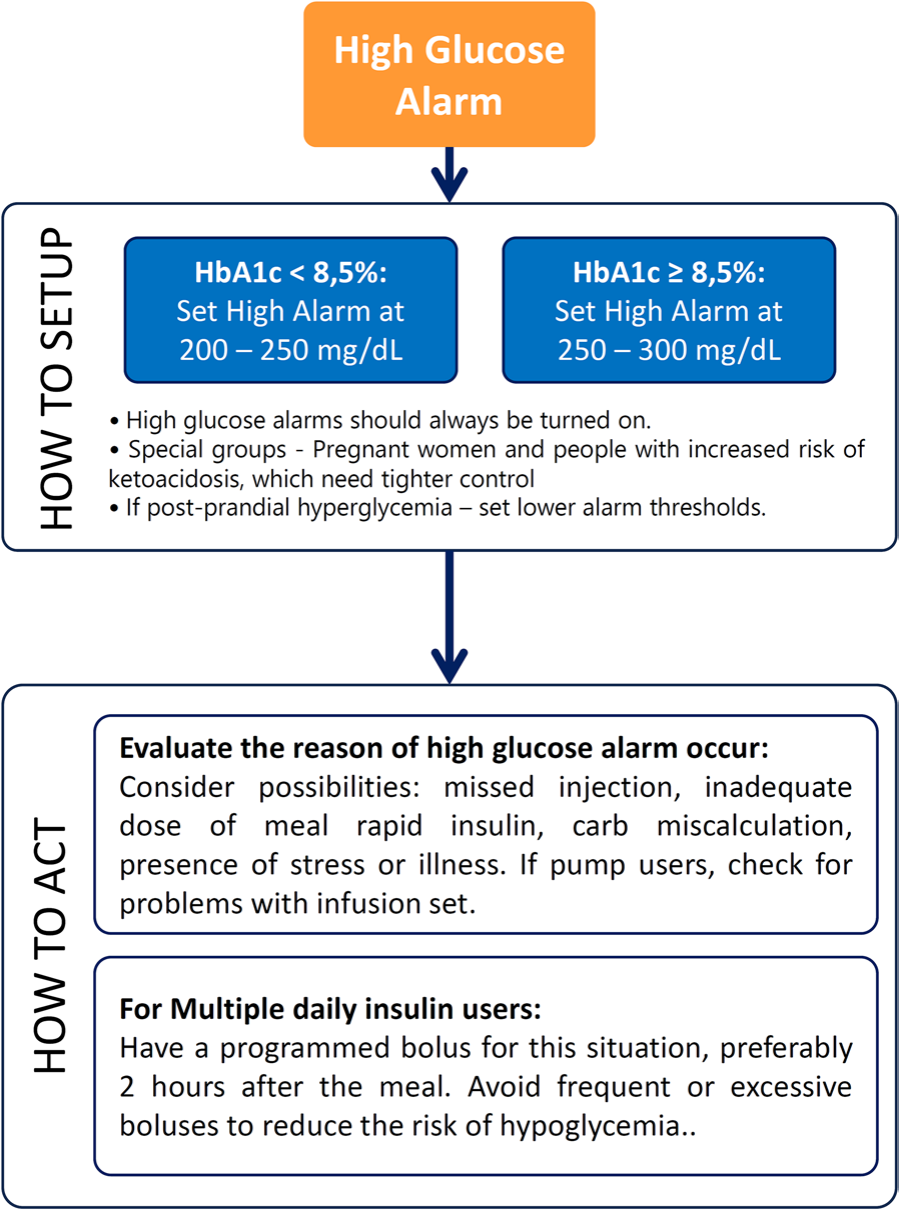
High glucose alert threshold. Based on reference [51]

**Fig. 3:**
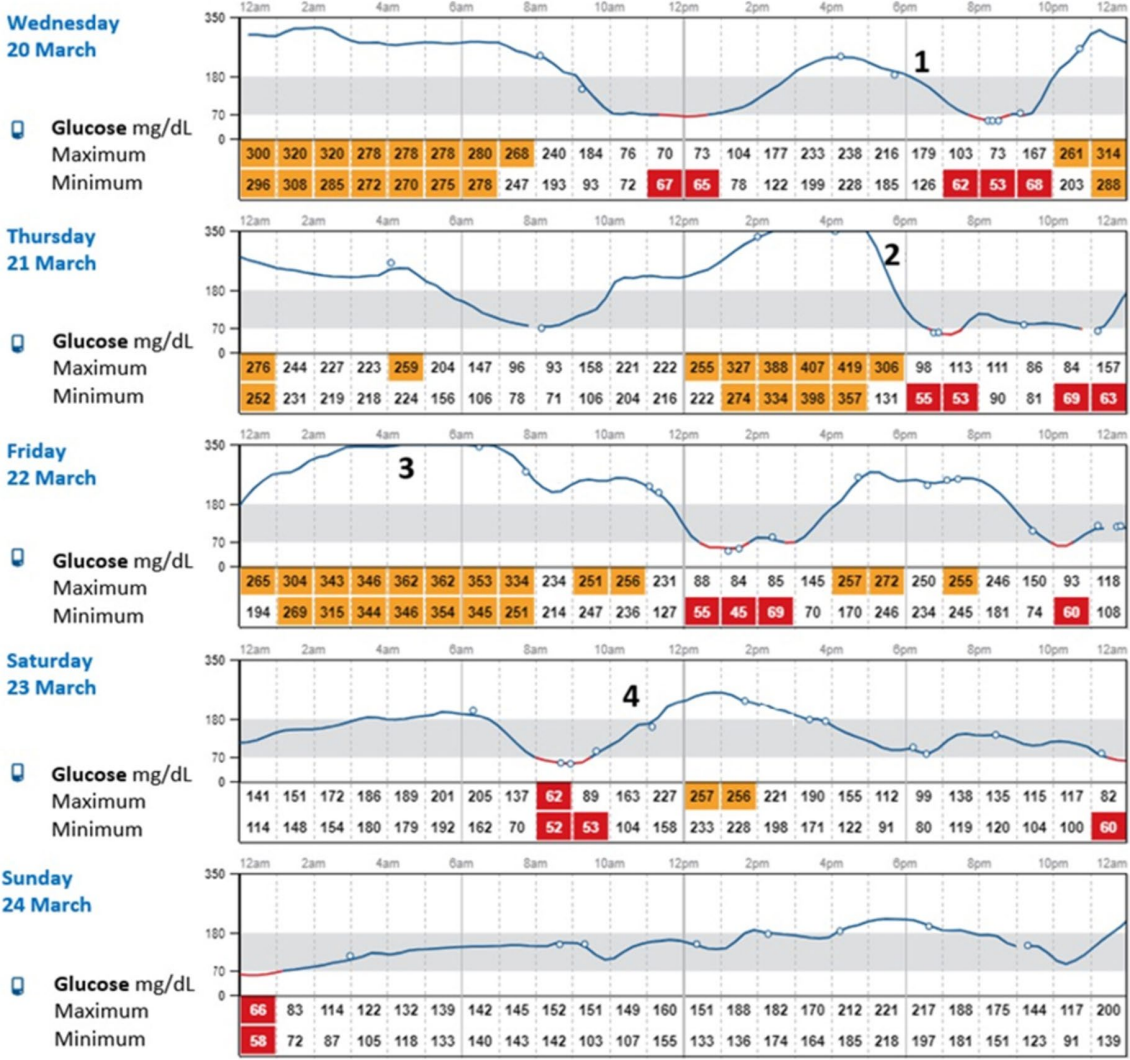
Practical tips about high glucose alert thresholds. 1. Alarms set to usual postprandial glucose levels can lead to overcorrection of insulin (stacking) and lead to hypoglycemia→try to avoid alarms at usual post-meal levels. **2**. Use alarms for the early detection of high glucose levels. **3.** Use alarms to detect very high glucose levels. **4.** Set for lower levels can lead to notification at every meal. Based on references [50, 74]

Regarding the level to set the alarm (Fig. 2) [51], it should be noted that the alarms are switched off by default and when activating them, the purpose of the alarms should be clearly explained to the subject. Thus, the level of threshold should be used to detect high or very high glucose levels, but not usual post-meal glucose levels, since this could lead to alert after each meal, which can increase diabetes burdens, or can lead to overcorrection of insulin. When a high glucose alert occurs, several factors should be evaluated. Was an insulin dose missed? Was the rapid-acting insulin dose for the meal insufficient? Is illness or stress contributing? For insulin pump users, could there be an infusion set failure? Was the carbohydrate intake miscalculated? Before correction, consider the insulin-to-carb ratio and remember that rapid-acting insulin takes 30–60 min to start lowering glucose and remains active for about 4 h. Therefore, after administering a correction dose, glucose levels should be rechecked in 2 h to assess the trend. Then, after correction, the alert may be modified to a more appropriate level. Some practical tips about high glucose alert thresholds are shown in Fig. 3 [51].

### Pregnancy

There are no studies directly addressing the benefits of each hypoglycemia or hyperglycemia alarm range during pregnancy. However, during this phase, glycemic control must be strict, which can increase the risk of hypoglycemia. The target range established during pregnancy is 63 to 140 mg/dL [58]. Values close to this target can be programmed as hypoglycemia and hyperglycemia alarm limits, respectively. More stringent values can be established in individual cases if there is adequate and careful guidance on how to proceed in the event of hypoglycemia or hyperglycemia alarms. Excessive, repeated, and inappropriate use of insulin to correct mild hyperglycemia outside of meals during pregnancy can lead to severe iatrogenic hypoglycemia. However, strict hyperglycemia alarms during this phase can be used to guide behavior changes, such as food choices and physical activity. Alarm limits can be readjusted throughout pregnancy on an individual basis [51].

## Flash glucose monitoring system with optional alarms: evidence in the management of diabetes

The FreeStyle Libre device, a first-generation intermittently scanned continuous glucose monitoring (isCGM) system, was released worldwide in 2014 without glucose alarms. An updated version, the FreeStyle Libre 2 system, was launched in 2018, featuring alarms for hypoglycemia and hyperglycemia [59]. More recently, in 2024, FreeStyle Libre 2 Plus sensor had its accuracy improved, with MARD of 8.2% in adults and 8.1% in ≥ 2 years old children and adolescents, with an additional day with 15-day sensor wear [60].

FreeStyle 2 sensor has a high accuracy, with a mean absolute relative difference (MARD) of 9.2% in adults and 9.7% in children and is approved for individuals aged ≥ 4 years [61]. In 2024, FreeStyle Libre 2 Plus sensor had its accuracy improved, with MARD of 8.2% in adults and 8.1% in children and adolescents ≥ 2 years old, with additional day with 15-day sensor wear [60]. Both sensors (FreeStyle Libre 2 and FreeStyle Libre 2 Plus) meet the rigorous performance requirements for being considered as an integrated CGM (iCGM) [32]. While the Freestyle Libre 2 Plus is considered an isCGM when handled with a reader, using with the FreeStyle LibreLink on a compatible smartphone enables real-time glucose readings transmission, effectively turning the sensor into an rtCGM. Glucose data from the FreeStyle LibreLink app could be shared automatically to healthcare professional platform [62], or even family member and caregivers [63]. FreeStyle Libre 2 system alarms are optional, and individuals can modify their low and high glucose thresholds, according to the clinical characteristics of subjects, allowing the individualization in the management of diabetes [56].

Several studies have demonstrated the benefits of flash glucose monitoring system with optional alarms. In a parallel-group, multicenter, randomized, controlled trial with 156 population with T1D and HbA1c 7.5–11.0% (mean age 44 years, mean duration of diabetes 21 years), compared to the own blood glucose levels monitoring with fingerstick testing (usual care), CGM with alarms was associated with a significant reduction in HbA1c levels at 24 weeks (adjusted mean between-group difference, − 0.5%; 95% CI − 0.7% to − 0.3%; P < 0.001). In addition, the time per day that the glucose level was in the target range was 9.0% higher or 130 min longer with CGM with alarms. Furthermore, time spent in a hypoglycemic state was also 3.0% lower or 43 min shorter with CGM compared to the control group [36].

In a prospective, observational study involving 47 children and adolescents with T1D, switching from the FreeStyle Libre to the FreeStyle Libre 2 with optional alarms for 14 days resulted in a 5% increase in time in range (TIR) (from 62.5% to 67.8%). Improvements were also observed in quality of life and reductions in time spent in hypoglycemia, the number of weekly hypoglycemic events, and the coefficient of variation. Notably, the use of alarms did not negatively impact the duration or quality of sleep for either children or their parents [64]. Other study that investigated the impact of switching from FreeStyle Libre to FreeStyle Libre 2 system in 38 adults with T1D (mean age 33.7 years) showed that after only 4 weeks of switching, TIR significantly increased from 52.8 to 57.0%, and time below range significantly decreased from 6.2 to 3.4%, as well as time < 54 mg/dl and coefficient of variation. The benefits were higher among those subject with more time in hypoglycemia at baseline. Importantly, treatment satisfaction improved, and concern of hypoglycemia decreased [42]. In a prospective observational study with 672 adults with type 1 diabetes, after 12 months of switching from FreeStyle Libre to FreeStyle Libre 2, time below range decreased by 1.0% and a high alert use was independently associated with an increase in TIR of ≥ 5% [65].

In another study that included 108 subjects with T1DM (mean age 58 years; mean diabetes duration 25 years) and fear of hypoglycemia, prone to hypoglycemia unawareness, and/or experiencing severe hypoglycemia while using FreeStyle Libre system, switching to FreeStyle Libre 2 with individually-programmable low glucose alarms translated into a reduction in time below range < 70 mg/dl from 4.5% to 2.3% (p < 0.001), time below range < 54 mg/dl from 1.4% to 0.3% (p < 0.001) and coefficient of variation from 39.4% to 37.9% (p < 0.001) at 12 weeks [41]. Moreover, those subjects at risk for hypoglycemia showed a significant decrease in the incidence of hypoglycemia, and the satisfaction of individuals with hypoglycemia alarms was high [41]. A cross-sectional study of 873 subjects with T1D that used the FreeStyle Libre 2 system showed that the use of hypoglycemia alarms reduced TBR and glucose variability, but with a shorter TIR. Additionally, the hyperglycemia alarms effectively reduced hyperglycemia and GMI [39]. In cross-sectional survey targeted people diagnosed with type 1 or type 2 diabetes requiring multiple daily injections of insulin as well a higher proportion of FreeStyle Libre 2 system users with optional alarms reported a greater satisfaction with their devices compared to the use of isCGM without alarms [66]. In another study performed in people with T1D, there were significant improvements in TIR, time below range, time above range, GMI, coefficient of variation and mean glucose 3 months after switching to FreeStyle Libre 2 from FreeStyle Libre. Remarkably, the mean number of daily scans with the FreeStyle Libre 2 was higher compared with the previous one, and this was the consequence of the use of alarms by the users, as they become more aware about daily changes in glycemic levels and the actions to be taken accordingly [67].

Other CGM with glucose alarms features also demonstrate clinical outcomes, with improvement of TIR and hypoglycemic events with switch from isCGM without alarms to rt-CGM with glucose alarms [68, 69]. Observational studies also show significant reduction in the duration of hypoglycemic excursions when low glucose alarm was defined; however, overtreating hypoglycemia may have resulted in a marginally significant increase in the frequency of hyperglycemic excursions [70].

## Educational aspects of glucose alarms

Continuous glucose monitoring, when coupled with education, follow-up, and support, can improve the lives and health of people with diabetes. It is essential to provide people with diabetes and their caregivers with initial and ongoing education and training, whether in person or remotely. Additionally, there should be continuous assessments of their technique, results, and ability to use data, including uploading and sharing data (if applicable), to monitor and adjust therapy. Those with more education regarding device use have better outcomes [8], and studies have demonstrated the efficacy of CGM education regarding improved glycemic outcomes and increase knowledge [71–73]. CGM-specific education has the potential to increase utilization of and response to alerts and alarms [44]. Table 1 illustrates relevant topics when initially configuring glucose alarms.

**Table 1:** Educational aspects when initiated glucose alarms

Education: Explain the importance of the alarms and how they can help in the management of diabetes. Offer continuous training on the use of CGM and interpretation of data
Psychological Support: Address symptoms and concerns related to alarms, helping individuals adapt to the use of CGM
Regular Reviews: Conduct periodic reviews of glucose data and adjust alarms as necessary. Use data reports to identify patterns and adjust alarm thresholds in a personalized manner
Gradual approach: Encourage a gradual approach to set up glucose alarms, especially for those who are initially resistant
Alarms for the user vs. alarms for the caregiver: Some systems, such as FreeStyle Libre 2, offer the possibility for another person (caregiver) to receive the alarms on their smartphone. The option to program alarms independently for the user and caregiver should be provided to avoid “alarm fatigue,” which is common among adolescents, especially in school. Since the occurrence of alarm fatigue has been significantly reduced by improving the accuracy of CGM, a gradual approach is needed, starting with the most important alert functions for that subject, and/or deactivating the caregiver’s alarm instead of the individual, if desired. Alarms can also be set to different values for the user and caregiver

Based on reference [51]

## Conclusions

CGM systems with optional alarms, like FreeStyle Libre 2 system has demonstrated the ability to reduce hyperglycemia and severe hypoglycemic events, leading to improvements in time in range (TIR) and quality of life of individuals. Optimal setting of low and high glucose alerts should be individualized to maximize efficacy and safety while minimizing 'alarm fatigue' caused by excessive notifications. When alarms occur, their underlying causes should be investigated to enable appropriate corrections and adjustments.

## Data Availability

No datasets were generated or analysed during the current study.
